# Unsupervised classification of plethysmography signals with advanced visual representations

**DOI:** 10.3389/fphys.2023.1154328

**Published:** 2023-05-23

**Authors:** Thibaut Germain, Charles Truong, Laurent Oudre, Eric Krejci

**Affiliations:** ^1^ Université Paris Saclay, Université Paris Cité, ENS Paris Saclay, CNRS, SSA, INSERM, Centre Borelli, Gif-sur-Yvette, France; ^2^ Université Paris Cité, Université Paris Saclay, ENS Paris Saclay, CNRS, SSA, INSERM, Centre Borelli, Paris, France

**Keywords:** respiration, breathing, dynamic time warping (DTW), clustering, machine learning

## Abstract

Ventilation is a simple physiological function that ensures the vital supply of oxygen and the elimination of CO_2_. The recording of the airflow through the nostrils of a mouse over time makes it possible to calculate the position of critical points, based on the shape of the signals, to compute the respiratory frequency and the volume of air exchanged. These descriptors only account for a part of the dynamics of respiratory exchanges. In this work we present a new algorithm that directly compares the shapes of signals and considers meaningful information about the breathing dynamics omitted by the previous descriptors. The algorithm leads to a new classification of inspiration and expiration, which reveals that mice respond and adapt differently to inhibition of cholinesterases, enzymes targeted by nerve gas, pesticide, or drug intoxication.

## 1 Introduction

Measurement of respiratory function in conscious, spontaneously breathing animals is essential in different settings such as studying drug effects on the respiratory system (Murphy, 2002), monitoring mouse models of human diseases (Willmann et al., 2017) or evaluating airway irritant molecules (Vijayaraghavan et al., 1993). Plethysmography methods are commonly used to record respiration. Several plethysmographs exist whole-body [WBP, Bartlett and Tenney (1970)], dual-chamber [DCP, Hoymann (2012)], head out-of-body [HOP, Vijayaraghavan et al. (1993)], and the choice between them is based on a trade-off between invasiveness and accuracy of measurement (Bates and Irvin, 2003).

For WBP, the mouse is placed in near-natural conditions: it is a large box where the mouse is not restrained and can move freely. Changes in pressure or flow within the chamber are measured over time, reflecting changes of volume, humidity and temperature of air entering and leaving the lung. Flow or differential pressure recordings allow the computation of the respiratory frequency and the volumes exchanged. Nevertheless, lung function is poorly measured, and the reproducibility of the experiment depends on many environmental parameters (Bates and Irvin, 2003; Bruggink et al., 2022).

The DCP consists of two sealed compartments where the animal’s head is in one compartment and its body in the other. The mouse is constrained in a tube with the nose pointing into the nasal compartment (respiration is primarily nasal in mice) (Mailhot-Larouche et al., 2018). This device allows for independent monitoring of the nasal airflow and the airflow caused by the thoracic movements of an animal. DCP is a relevant approach to assessing the ventilatory mechanics of the respiratory system, and it provides information on ventilatory and lung function (Hoymann, 2012). As the mouse is constrained, it limits the duration of respiration recording to less than 1 h. As an alternative, the HOP uses only the thoracic compartment imposing less constraint on the mouse (Vijayaraghavan et al., 1993). Recordings from DCP and HOP directly reflect the air inhaled and exhaled during respiration. These methods have been used for several decades to monitor changes in mouse respiration caused by airborne chemicals on the airways (Vijayaraghavan et al., 1993) and have been improved to limit air leakage from collar (Bruggink et al., 2022).

Respiration airflows are used to compute respiratory cycle descriptors like inspiration/expiration duration, air volume inhaled/exhaled, or respiratory frequency [IXO2 software, emka TECHNOLOGIES, Mailhot-Larouche et al. (2018)]. These descriptors are essential to quantify respiratory exchanges; nevertheless, they only reveal part of the information contained in the respiration airflow.

Recently, a new machine learning-based method attempted to incorporate the missing information by extracting respiratory cycle patterns from flows recorded in WBP (Sunshine and Fuller, 2021). They used principal component analysis and a clustering algorithm to group common respiratory cycle patterns. The groups reveal variations in the temporal appearance of the flows that are not detectable with standard analysis of respiratory rate and tidal volume. Because each group had physiological significance, they could track significant changes over time. However, the different categories cannot be associated with physiological alterations or adaptations because the signals recorded in WBP mix too many parameters (volume/temperature/humidity).

It is well established that DCP or HOP is much more accurate for studying respiratory physiology than WBP. Nonetheless, descriptors inferred from the nasal or thoracic airflow also miss meaningful information to describe breathing dynamics adequately. In this study, we propose a new approach to classify the different respiratory behaviors from signals recorded with DCP or HOP. The method relies on a robust algorithm to identify the beginning of inspiration and expiration phases. As a difference with (Sunshine and Fuller, 2021) which studies the respiration cycle, we independently study inspiration and expiration. Our method is based on machine learning tools for time series. It uses a K-Means clustering algorithm and the well-established Dynamic Time Warping (DTW) distance. DTW compares the shape of time series independently of time fluctuations. This property is particularly interesting for handling inter-individual variability. In contrast to (Sunshine and Fuller, 2021), we assess the similarity between inspiratory or expiratory cycles directly from their shapes rather than from learned features. It allows a more robust and interpretable study of respiratory behaviors and dynamics. To evaluate the relevance of this new method, we exploited part of the recordings from a previous experiment of our group (Nervo et al., 2019), where we studied the consequences on respiration of partial deficits of acetylcholinesterase (AChE) and its inhibition. AChE normally destroys acetylcholine (ACh) in synapses of the nervous systems (central and peripheral) and skeletal muscles. Inhibition of this enzyme results in respiratory arrest, which may have multiple origins (Stone, 2018).

## 2 Methods

### 2.1 Background

**FIGURE 1:**
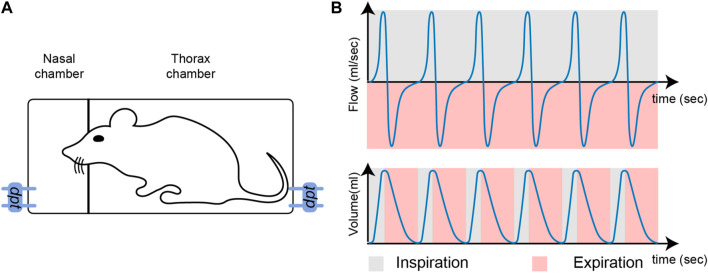
**(A)**: Illustration of a double-chamber plethysmograph. The term *dpt* stands for differential pressure transducer which measures the pressure in each compartment, the pressure then being converted to flow. **(B)**: Nasal airflow (top) and lung volume (bottom). During inspiration, airflow is positive (grey) and during expiration, airflow is negative (pink).

With a Double Chamber Plethysmograph (DCP), the nasal airflow induced by breathing is tracked through the head compartment (Hoymann, 2012), as illustrated on Figures 1A. The flow is expressed in *ml*.*s* ^−1^, and is a positive quantity for inspiration, and a negative quantity for expiration. The detection of inspiration or expiration start times cannot be done accurately from the nasal airflow due to biological phenomena such as coughing or vocalizing (Bates and Irvin, 2003). As an alternative, the lung volume, which is obtained by integration of the nasal airflow, offers more robust properties for such detection (Vijayaraghavan et al., 1993). Intuitively, the lung volume fluctuates successively from being empty (inspiration start time: *t*_*in*_) to being full (expiration start time: *t*_*out*_). These states correspond to local minima and maxima on the volume, which are easy to track with automated procedures. The characterization of breathing phases is illustrated in Figures 1B.

### 2.2 Overview of the method

The method is composed of three main steps:

1. Detection of the respiratory cycles and extraction of the inspiration/expiration sequences,
2. Computation of the reference sequences through an unsupervised clustering procedure,
3. Characterization and symbolization of recordings based on the extracted reference sequences.

**FIGURE 2:**
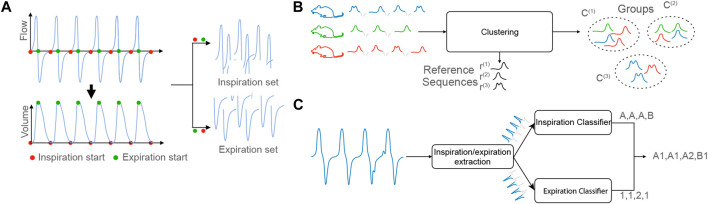
**(A)**: Step 1, Detection of the respiratory cycles and extraction of the inspiration/expiration sequences. **(B)**: Step 2, Computation of the reference sequences. The *C* ^(*i*)^ denote the clusters and **r** ^(*i*)^, the reference sequences. **(C)**: Step 3, Characterization and symbolization of a recordings.

Step 1: Detection of the respiratory cycles and extraction of the inspiration/expiration sequences. The first step of the process consists in extracting the respiratory cycles from the input data. Each cycle is composed of two phases: inspiration and expiration. A segmentation algorithm isolates the two periods. Simplistically, given a raw signal **s**, the first step of our method outputs a set of inspiration sequences $\left\{s_{in}^{\left(1\right)},\ldots ,s_{in}^{\left(N_{s}\right)}\right\}$ and a set of expiration sequences $\left\{s_{\mathrm{out}}^{\left(1\right)},\ldots ,s_{\mathrm{out}}^{\left(N_{s}\right)}\right\}$ , where *N*_*s*_ is the total number of cycles observed in the original signal **s**. Figures 2A illustrates the detection and extraction process of inspiration/expiration.

Step 2: Computation of the reference sequences. The second step consists in computing a small number of reference sequences from the sets of inspiration/expiration sequences. The reference sequences represent groups of sequences with common properties to highlight typical inspiration/expiration behaviors. To that aim, the clustering algorithm K-means is combined with the measure of fit Dynamic Time Warping (DTW), which computes the similarities between sequences of potentially different lengths. The output of this step is a set of inspiration reference sequences $\left\{r_{\mathrm{in}}^{\left(1\right)},r_{\mathrm{in}}^{\left(2\right)},\ldots \;\right\}$ and a set of expiration reference sequences $\left\{r_{\mathrm{out}}^{\left(1\right)},r_{\mathrm{out}}^{\left(2\right)},\ldots \;\right\}$ . Figures 2B illustrates the computation process of reference sequences in the case of inspiration.

Step 3: Characterization and symbolization of recordings. The objective is to automatically characterize a recording **s**′ using the reference sequences extracted in Step 2. To that end, the signal is first segmented through the procedure described in Step 1. Then, each of the *N*_*s*′_ inspiration/expiration sequences present in **s**′, is assigned a symbol which represents the reference sequence that is closest considering the measure DTW. This procedure results in a symbolic representation of **s**′, where each respiratory cycle is replaced by a symbol composed of a letter (which specifies the type of inspiration sequence observed) and a number (which specifies the type of expiration sequence observed), Figures 2C illustrates the process of building a symbolic representation.

### 2.3 Detection of the respiratory cycles and extraction of the inspiration/expiration sequences

As mentioned previously, nasal airflow suffers from noise, making current inspiration and expiration phases detection methods unreliable. Inaccurate detection then leads to biased descriptors and eventually to false experimental conclusions. To address this challenge we propose an algorithm that looks for local minima and maxima of the lung volume. Let **s** denote a nasal airflow signal.

First the lung volume **v** is computed from the nasal airflow **s**. This can be done by robust numerical integration:

$$v_{t}≔\left(\overset{t}{\underset{u=1}{\sum }}s_{u}\right)-\left(\hat{a}t+\hat{b}\right)$$ (1)

where $\hat{a},\hat{b}\in R$ are such that *∑*_*t*_ *v*_*t*_ = 0 and *∑*_*t*_ *tv*_*t*_ = 0. The affine function $t\to \hat{a}t+\hat{b}$ removes the linear trend appearing during the integration process.

Next, the inspiration start times *t*_*in*_ and the expiration start times *t*_*out*_ are identified using a peak-searching procedure that detects local minima (respectively maxima), of the nasal volume signal **v**. To ensure an alternation between inspiration and expiration, the algorithm first searches for all local minima (corresponding to the starts of the inspirations) and then searches for the maximum between two consecutive local minima. The algorithm that detects local minima/maxima is described in Supplementary Appendix S1. Once all inspiration/expiration start times *t*_*in*_ and *t*_*out*_ are extracted, the original nasal airflow signal **s** is split into a set of inspiration sequences $\left\{s_{in}^{\left(1\right)},\ldots ,s_{in}^{\left(N_{s}\right)}\right\}$ and a set of expiration sequences $\left\{s_{\mathrm{out}}^{\left(1\right)},\ldots ,s_{\mathrm{out}}^{\left(N_{s}\right)}\right\}$ , where *N*_*s*_ is the total number of cycles observed in the original signal **s**.

### 2.4 Computation of the reference sequences

Provided a set of inspiration/expiration sequences, we now aim to compute *K* reference sequences that represent typical respiratory behaviors. In the following sections, $X=\left\{x^{\left(1\right)},\ldots ,x^{\left(N\right)}\right\}$ represents a set of sequences (either inspiration or expiration) of potentially different durations.

#### 2.4.1 Clustering algorithm

**FIGURE 3:**
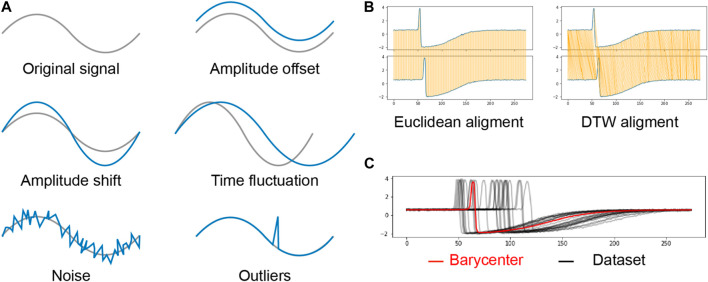
**(A)**: Undesirable sequence distortions. The grey line is the original signal and the blue line is the distorted signal. **(B)**: Difference between Euclidean alignment and DTW alignment. The compared sequences are in blue, and the orange lines represent the point-wise matching between the two sequences in the Euclidean case and the DTW case. **(C)**: Representation of a barycenter computed through our method BS-DBA. The learned barycenter is in red, and the observations are in black.

The *K* reference sequences from the set X are computed with the well-known unsupervised clustering procedure called K-Means (Kaufman and Rousseeuw, 2009). This algorithm creates *K* non-overlapping groups (or clusters) $\left\{C^{\left(1\right)},\ldots ,C^{\left(K\right)}\right\}$ of sequences with common properties. Roughly, K-Means is a two-step iterative refinement technique that assigns each sequence to the closest current centroid and then updates each centroid with regard to the new assignments. A centroid is a reference sequence **r** ^(*i*)^ which corresponds to the average sequence of the cluster $C^{\left(i\right)}$ . Two crucial ingredients of the K-means algorithm are the measure of fit that is used to assign each sequence to a cluster and the procedure used to compute the reference sequence of each cluster. Although most publications usually use the Euclidean distance, it is not possible in our context since the sequences to cluster do not have the same duration. Also, the measure of fit must be invariant to some sequence properties: amplitude offset, amplitude shift, time fluctuation, noise and outliers. Visual representation of each distortions are presented in Figures 3A.

The main steps of our clustering approach are:

1. The input sequences are pre-processed by taking the z-normalized sequences.
2. The clustering algorithm is initialized using the K-Means++ algorithm (Arthur and Vassilvitskii, 2007).
3. Sequences are assigned to a cluster according to the Dynamic Time Warping (DTW) measure of fit.
4. The reference sequences are computed using the Batch Stochastic DTW Barycenter Averaging (BS-DBA) procedure.
5. Step 2 and 3 are repeated until a certain stopping criterion is met.

For our experiment, the stopping criterion corresponds to 10 iterations of steps 2 and 3.

#### 2.4.2 Pre-processing

During the pre-processing step, all sequences are first centered to zero mean and scaled to unit variance (z-normalization):

$${\tilde{x}}_{t}=\frac{x_{t}-\bar{x}}{\sigma_{x}}$$ (2)

where $\bar{x}$ and *σ*_*x*_ are respectively the average and the standard deviation of **x**. The pre-processing step allows being invariant to amplitude offset and amplitude shift.

#### 2.4.3 Dynamic time warping

At each iteration, the K-Means algorithm assigns each sequence to the nearest centroid. The distance is computed using DTW (Berndt and Clifford, 1994). DTW is commonly used in times-series data-mining (Fu, 2011; Esling and Agon, 2012). Intuitively, DTW considers as very similar (the distance is close to zero) two sequences of a given phenomenon occurring at different speeds. This property is particularly interesting for our problem since some mice may breathe in or exhale faster than others. To do this, DTW finds an optimal match between a query sequence and a referent sequence by locally stretching or contracting the time axis of the query sequence. The DTW measure produces the squared Euclidean distance between the aligned time series.

This measure is invariant to temporal fluctuation and can compare sequences of different duration. Considering two sequences $x\in R^{m}$ and $y\in R^{n}$ , the computation of the DTW measure is done in *O*(*mn*) in time and space using dynamic programming. In its original form, the DTW measure is sensitive to noise and outliers. Such distortions can lead to pathological alignments with unrealistic time dilations. To avoid such alignments, we use the Sakoe-Chiba constraints which impose that the dilations are smaller than a given duration (Sakoe and Chiba, 1978).

Figures 3B shows the difference between the linear mapping of the Euclidean distance and the nonlinear mapping of the DTW distance. A mathematical definition of DTW is given in Supplementary Appendix S2.

#### 2.4.4 Time-series averaging

Finding an average sequence is an important sub-routine of K-Means algorithm. Indeed, the quality of each cluster is highly dependent on the quality of its centroid (Aghabozorgi et al., 2015). At each iteration, all sequences in the data set X are assigned to their closest centroids $\left\{r^{\left(1\right)},\ldots ,r^{\left(N\right)}\right\}$ . Then, each centroid is updated by computing the average sequence based on the new assignment.

For any set of sequences $X^{\prime }\subset X$ , the average sequence, with respect to the DTW, is the solution of the following optimization problem:

$$\underset{y\in R^{L}}{\mathrm{arg min}}\underset{x^{\prime }\in X^{\prime }}{\sum }{\text{DTW}}^{2}\left(y,x^{\prime }\right)$$ (3)

where *L* > 0 is the average duration of the sequences in $X^{\prime }$ .

Accurately and efficiently solving Expression 3 is not trivial (Niennattrakul and Ratanamahatana, 2007; Jain, 2019). Traditional averaging methods cannot deal with the non-linear mapping between sequences of potentially different duration and several algorithms have tried to solve this issue (Petitjean et al., 2011; Morel et al., 2018). A recent work (Schultz and Jain, 2018) uses the subdifferentiability property of the optimization function to develop a stochastic subgradient descent algorithm (S-DBA). For a trade-off between accuracy and speed, we implemented a batch version of S-DBA called BS-DBA. Figures 3C illustrates the result of averaging a time series data set using BS-DBA. Supplementary Appendix S3 provides details on the subdifferentiability of the optimization function and the implementation of BS-DBA.

### 2.5 Characterization and symbolization of recordings

From a recording **s**′, we first perform the segmentation process described in Section 2.3 in order to extract the inspiration/expiration sequences. Then, we use a 1-NN (nearest neighbor) algorithm to assign each sequence to the reference sequence, which is the closest to it, in the sense of the DTW measure.

To avoid incoherent symbols, some inspiration/expiration sequences are treated as outliers if their distance to their reference sequence is higher than a threshold. The threshold is different for each reference sequence. It corresponds to the *α*-quantile of the distance distribution observed within the reference sequence cluster during the learning step. By default we choose the threshold value *α* = 0.95.

This procedure yields a symbolic representation of **s**′, where each respiratory cycle is replaced by a symbol composed of a letter (which specifies the type of inspiration) and a number (which specifies the type of expiration).

### 2.6 Connection with ventilation pattern descriptors

Most ventilation pattern descriptors are computed with algebraic formulas based on a cycle segmentation of airflow using IOX2 software from emka TECHNOLOGIES (Mailhot-Larouche et al., 2018). The algorithm to extract inspiration and expiration sequences presented in Section 2.3 can be used as a preprocessing step to compute such descriptors with more precision.

In the present work and for the purpose of validation, we have used four descriptors:

•**Inspiratory/Expiratory Time (Ti/Te,** ***s*** **):** Duration of inspiration/expiration.
•**Nasal Inspiratory/Expiratory Volume (NIV/NEV,** ***ml*** **):** Volume of air in/out during inspiration/expiration.

## 3 Data and experiment

### 3.1 Data origin

We applied our methodology to a subset of data from experiments that aimed to understand and evaluate how cholinesterase (ChE) inhibitors affect mice respiration with partial deficit in AChE (Nervo et al., 2019). Acetylcholine (ACh) is a well-known neurotransmitter in the central and peripheral nervous systems. It is also found at the neuromuscular junction (NMJ). ACh in synapses is hydrolyzed by acetylcholinesterase (AChE). ACh is also used by numerous non-neuronal cells to communicate (Grando et al., 2015). Inhibition of ChE changes the dynamic of ACh and thus may modify respiration at different physiological levels. To better understand the mechanisms, we have recorded the nasal and thoracic airflow of mice with different partial AChE deficits induced by injection of physostigmine, an inhibitor of ACHe, using a Double Chamber Plethysmograph (DCP), (Mailhot-Larouche et al., 2018). We recorded nasal and thoracic airflow from control mice (WT mice), PRiMA KO mice [PRiMA mice: AChE deficiency in cholinergic neurons of the brain and peripheral nervous systems (autonomic and enteric)] (Farar et al., 2012); muscle KO mice (AChE1iRR; absence of AChE in skeletal muscles); ColQ KO mice (ColQ mice: no AChE anchoring in muscles and some tissues) (Bernard et al., 2011).

As described in (Nervo et al., 2019), mice of different genotypes were exposed as follows:

1. Phase 1: The mouse is placed in a DCP for 15 or 20 min to serve as an internal control.
2. Phase 2: The mouse is removed from the DCP and injected with physostigmine.
3. Phase 3: The mouse is placed back into the DCP, and its nasal flow is recorded for 35 or 40 min.

### 3.2 Experiment

In order to test our approach, we have run and evaluated the results of the following experiment:

1. Creation of a data set.
2. Extraction of training data set for inspiration/expiration.
3. Computation of inspiration/expiration referent sequences.
4. Symbolization of all signals in the data set.

Step 1: Creation of a data set. Our data set includes the nasal airflow recording of 32 different mice. Among all recordings available from Nervo et al. (2019), we have selected 8 mice for each genotype: WT, PRiMA, AChE1iRR, ColQ. All mice were exposed to the same inhibitor: physostigmine. All signals were recorded at 2,000 Hz and have been down-sampled to 250 Hz. By default, the double chamber plethysmograph includes a bandpass filter, whose band limits are 0.250 Hz and 35000Hz, which has not been modified.

Step 2: Extraction of training data set for inspiration/expiration. On average, a mouse’s respiratory cycle lasts about 0.3 s. The original data set contains approximately 350,000 cycles, and therefore computing reference sequences (Section 2.4) from the entire data set would have been time-consuming. Thus, for each recording, we extracted 1,800 cycles that were evenly selected in time. This subsampling corresponded to approximately 36 cycles per minute, resulting in a set of 57,600 cycles that were divided into an inspiration training data set and an expiration training data set.

Step 3: Computation of inspiration/expiration reference sequences. Referent sequences were computed according to the algorithm presented in Section 2.4. The hyperparameters are presented in the following section. The learning is based on the inspiration/expiration training data set.

Step 4: Symbolization of all signals in the data set. All recordings in the original data set are symbolized. The symbolization is based on the reference sequences learned from the training data sets.

### 3.3 Hyperparameters

The main parameters are presented below. Parameters for respiratory cycle detection have been set based on physician knowledge of the typical respiratory cycles. For the clustering algorithm, the number of clusters has been set arbitrarily and the Sakoe Chiba radius authorizes small dilatation.

• Respiratory cycle detection (Step 1):
  • Prominence: 0.03 mL
  • Window length: 2 s
  • Minimum inspiration/expiration duration: 0.05 s
  • Maximum inspiration/expiration duration: 2 s
• Clustering algorithm (Step 2, identical settings for inspiration and expiration):
  • Number of clusters: 5
  • Number of iterations for K-Means: 10
  • Sakoe Chiba radius: 0.01 s
  • Reference sequence length: 0.2 s
• Symbolization (step 3):
  • Quantile threshold: 0.95

A python implementation of the method is available https://github.com/thibaut-germain/DCP_Clustering.

## 4 Results

In this section, we summarized the complete pipeline of our method. It is composed of three main steps:

1. The first step consists in detecting of the respiratory cycles and extracting the inspiration/expiration sequences from the input data, Figure 2A.
2. The second step consists in computing a small number of reference sequences from the sets of inspiration/expiration sequences. The reference sequences represent groups of sequences with common properties to highlight typical inspiration/expiration behaviors, Figure 2B.
3. The third step consists in simplifying a recording using a symbolization based on the reference sequences extracted in Step 2, Figure 2C.

### 4.1 Categorization of the respiratory cycles

We first aim to categorize breathing cycles, inspirations, and expirations. The limits of inspiration and expiration are unambiguously defined from the volume obtained by integrating the flow (Vijayaraghavan et al., 1993). We define a referent cycle as the association between a referent inspiration and a referent expiration. Considering *K*_1_ referent inspirations and *K*_2_ referent expirations, there exist *K*_1_ *K*_2_ referent cycles. In order to compare them, we develop a map where each row corresponds to a referent inspiration and each column to a referent expiration. Each referent cycle is represented by an actual cycle selected as follows:

• Among the identically labeled cycles in the training database, we select the cycle whose cumulative DTW distance (DTW distance to the referent inspiration + DTW distance to the referent expiration) is the smallest.
• The respiratory cycle map can be displayed using either the nasal airflow or the nasal volume. In any case, the inspiration/expiration phases are matched, accordingly, to their attributed colors. For inspiration, the color scale goes from red to yellow; for expiration, it goes from blue to green.
• Inspiration/expiration referent sequences are ordered in increasing order according to the average duration observed in each group. Therefore, as the number/letter increases, the average inspiration/expiration duration is longer. Visually, lighter colors (yellow/green) correspond to longer duration.

**FIGURE 4:**
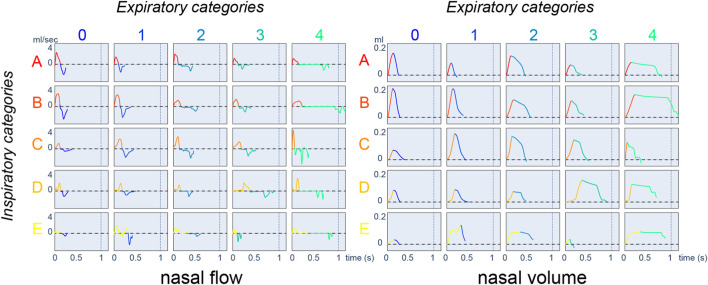
Respiratory cycle map displays with nasal airflow (*mL*.*s* ^−1^) on the left and nasal volume (*mL*) on the right. Positive flow corresponds to inspiration and negative flow corresponds to expiration.

In our experiment, we set the number of inspiration and expiration referent sequences to 5, as presented in Figure 4. Short duration cycles (A0, A1, B0, B1) are characterized by a nasal airflow of sinusoidal shape. All 25 of the resulting classes are used in the following sections to visualize and compare the respiratory cycles of mice of different strains before and after physostigmine injection.

### 4.2 Distribution of respiratory cycle categories

**FIGURE 5:**
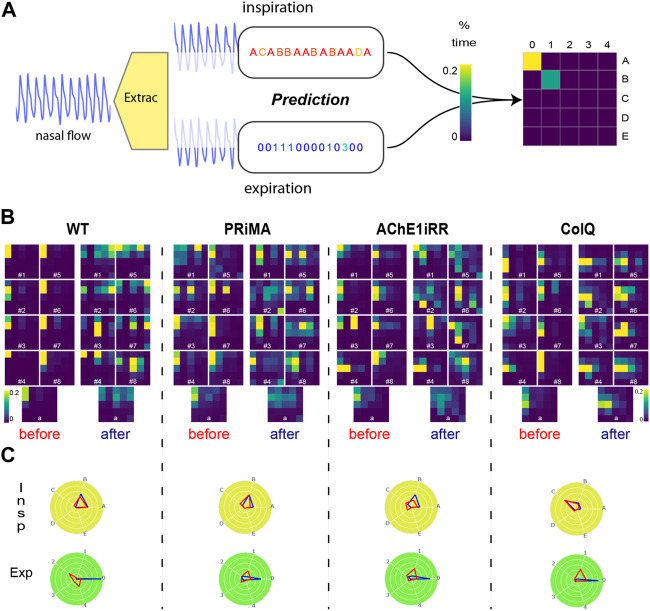
**(A)**: Respiratory Cycle map (RC map) built-up process. **(B)**: Respiratory RC maps: All RC maps are truncated at the threshold value of 20%. RC maps are grouped by genotype: WT, PRIMA, AChE1iRR, ColQ. For each genotype, the two left columns and the two right columns gathered RC maps respectively before and after physostigmine injection. Numbers on RC maps correspond to the mouse id. The bottom line corresponds to the average RC maps observed per genotype before and after drug injection. **(C)**: Average reference sequence polar plots: Polar plots are grouped by genotype. Inspirations are on the top, and expirations are on the bottom. The values on each angular axis correspond to the average percentage of time assigned to the associated reference sequence. The blue polygon corresponds to the values observed before injection, and the red polygon corresponds to the values observed after the injection.

In order to study the importance of each reference cycle for a given symbolic representation, we introduce a new visualization of the histogram that takes the form of a heat map. The respiratory cycle map (RC map) corresponds to a heat map where rows are inspiration symbols and columns are expiration symbols as presented in Figure 5A. Thus, each cell corresponds to a referent cycle, and its value is set to the percentage of time assigned to that specific referent cycle. To ease the study of less frequent referent cycles, we use a thresholded version of the respiratory RC map where all reference sequences that represent more than 20% of the total duration are assigned to the threshold value of 20%. A RC map provides a quick understanding of the dominant respiratory behavior of a mouse. In addition, RC maps can be aggregated over a population, allowing comparisons of a mouse’s respiratory behavior to the average behavior.

In Figure 5B, RC maps are grouped by genotype: WT, PRIMA, AChE1iRR, ColQ. For each genotype, the two left columns gathered RC maps before injection, and the two left columns gathered RC maps after injection. The bottom line corresponds to the average RC maps observed per genotype before and after drug injection.

In addition, we have created two conjoint polar plots, one for inspiration and one for expiration. Each angular axis corresponds to a referent sequence, and the value on each axis is equal to the percentage of time assigned to that specific referent sequence. These values are linked together to form a polygon. As for RC maps, the visualization can be done at the individual level or aggregated over a group of mice. This representation complements RC maps as it decorrelates inspiration from expiration, easing the study of both mechanisms independently as presented in Figure 5C.

### 4.3 Time line representation of respiratory cycle categories (bar codes)

Previous representations give an overview of the respiratory behavior of a mouse or a population. Nonetheless, they do not offer insights into the temporal evolution of a mouse’s respiratory behavior when facing a stressor. This evolution can be read from the symbolic representation with proper visualization.

**FIGURE 6:**
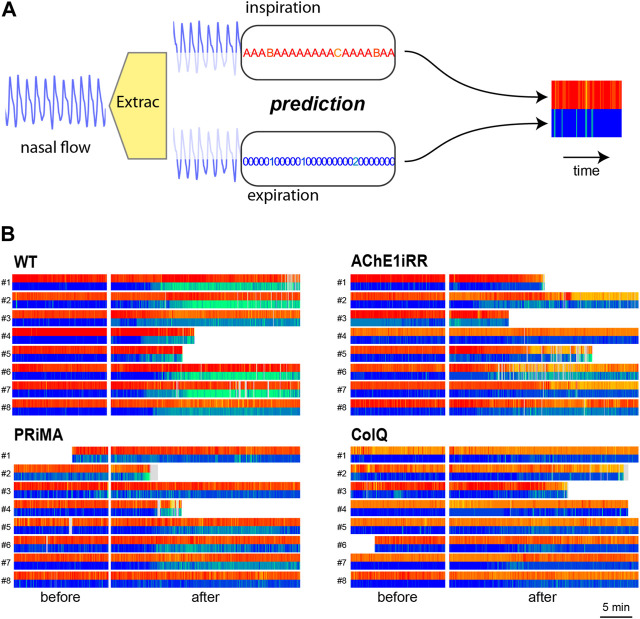
**(A)**: Respiratory bar codes built-up process.**(B)**: Respiratory bar codes are gathered by genotype: (top,left): WT, (bottom, left): PRIMA, (top,right): AChE1iRR, (bottom, right): ColQ. Numbers to the left of bar codes correspond to the mouse id. For each genotype, the left section corresponds to barcodes before drug injection and the right section to bar codes after injection. Grey areas in bar codes like mouse PRIMA-2 correspond to unpredictable cycles. Some experiments were shorter than others resulting in shorter bar codes.

To that aim, we construct a respiratory bar code for each mouse that includes the time information, as presented in Figure 6A. The respiratory bar code is composed of two lines, the upper line represents the inspirations, and the lower line representing the expirations. The central white area corresponds to the period of inhibitor injection, and the light grey area corresponds to unpredictable cycles. Each line is composed of rectangles whose color refers to the associated reference sequence and whose length is proportional to the duration of the associated respiratory cycle.

Figure 6B presents respiratory bar codes of all mice in the data set. They are gathered by genotype, and mouse identification numbers are on the left of the bar codes. For each genotype, the left section corresponds to bar codes before injection and the right section to bar codes after injection.

### 4.4 Statistical analysis of respiratory cycle categories

RC maps provide visual comprehension of the heterogeneity in breathing behaviors and changes due to the presence of a stressor. In complement to the visual presentation, we provide a statistical analysis that compares the breathing behaviors between genotypes and the breathing responses to the presence of a stressor.

The first statistical test compares the respiratory cycle distribution of AChE-deficient mice (PRIMA, AChE1iRR, ColQ) with that of control mice (WT). The null hypothesis is that the cohort of AChE-deficient mice has the same respiratory cycle distribution as the cohort of control mice. The alternative hypothesis is different respiratory cycle distributions.

The second statistical test compares the distribution of respiratory cycles for each genotype before and after drug injection. For the cohort of a given genotype, the null hypothesis is to have the same distribution of respiratory cycles before and after drug injection. The alternative hypothesis is different respiratory cycle distributions.

In both cases, we implemented a multiple testing scheme with a false discovery rate (FDR) correction of 5%, performing a Mann-Whitney U test for each type of respiratory cycle. Application of this test gives a map where each cell represents a type of respiratory cycle, with the row corresponding to the type of inspiration and the column to the type of expiration. A cell is colored black if the unit null hypothesis is rejected after FDR correction. In each cell, we also displayed the corrected *p*-value of the associated unit test.

**FIGURE 7:**
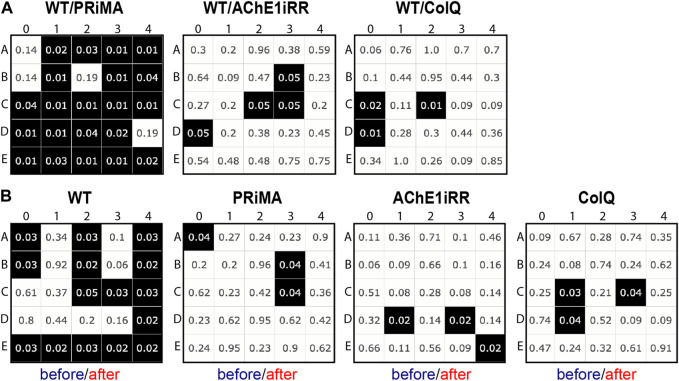
Multiple testing scheme with a false discovery rate (FDR) correction of 5%, performing a Mann-Whitney U test for each type of respiratory cycle. A cell is colored black if the unit null hypothesis is rejected after FDR correction and includes the corrected *p*-value of the associated unit test. **(A)**: Statistical tests comparing the distribution of respiratory cycles of control (WT) and AChE-deficient (PRIMA, AChE1iRR, COLQ) mice before drug injection. **(B)**: Statistical tests comparing the distribution of respiratory cycles before and after drug injection for each genotype.

All tests are rejected, Figure 7A, and the number of unit tests rejected at 5% is for WT vs. PRIMA: 21, WT vs. AChE1iRR: 4, WT vs. COLQ: 3. Similarly, all tests are rejected, Figure 7B, and the number of unit tests rejected at 5% is for WT: 15, PRiMA: 3, AChE1iRR: 3, COLQ: 3.

## 5 Discussion

This paper presents a new method to compare and quantify cyclic signals that may be particularly appropriate for biological investigations, such as respiratory signals. Rather than comparing cycles based on the ventilation descriptors, cycles’ shapes are compared to shape representations of most typical cycles. We will discuss the contributions and limitations of this new strategy by analyzing a part of recordings previously published (Nervo et al., 2019).

### 5.1 Inspiration and expiration classes fit respiratory physiological control

**FIGURE 8:**
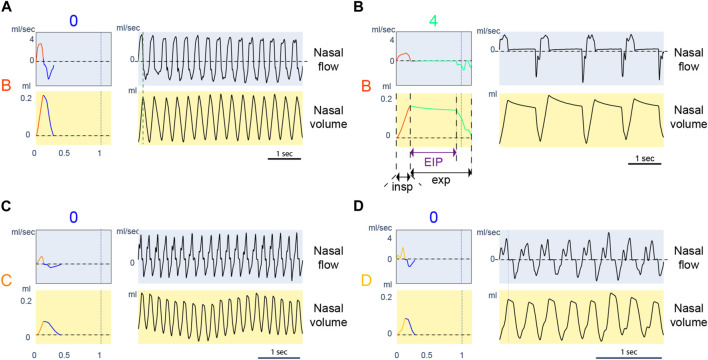
Examples of typical respiratory behaviors. For each panel, the left column represents the referent cycle, and the right column is an extract from a recording of up to 5 s where the reference cycle is repeated continuously. Charts with a blue background are expressed in nasal airflow, and charts with a yellow background are expressed in nasal volume. **(A)**: Referent cycle B0. **(B)**: Referent cycle B4. Inspiration, expiration, and end-inspiratory pause (EIP) duration are illustrated. **(C)**: Referent cycle C0. **(D)**: Referent cycle D0.

The classes learned with the new approach represent various respiratory profiles that carry biological meaning. We illustrate some respiratory profiles through their classes in Figure 8. The last 15 min before physostigmine injection represents mice’s baseline breathing behaviors. The control mice (WT) breathe with cycles of type A0 and B0. Figure 8A shows 5 consecutive seconds of a raw signal with respiratory cycles of B0. After injection of physostigmine, the inspiration classes (A and B) are not changed for the control mice (WT), as shown with the polar plot (Figure 5C). However, the expiration class changes from type 0 to type 2, 3, 4. Raw signals of 5 consecutive seconds of classes B4 are presented in Figure 8B. The profile of these classes shows a long pause when the lungs are inflated. They correspond to post-inspiratory pauses. They were analyzed in Nervo et al. (2019), and the authors quantified the duration of these pauses. The new approach captures significant respiratory behaviors making previous results apparent with the new representation: for control mice (WT), post-inspiratory pauses appear after inhibitor injection.

The approach also presents details about the inspiration dynamic of ColQ mice. Indeed, the cycles of ColQ mice before injection are grouped into types C0 and D0, which we present in Figure 8C, D. Inspiratory classes C and D are characterized by a nasal airflow that enters in two phases. The two phases in class D are distinctive. Compared to D, the separation between phases is less visible in C. The ColQ mouse is a model of congenital myasthenic syndrome with AChE deficit at the neuromuscular junctions. This mouse shows an impairment of motor control, which could be reflected during the motor control required for a smooth inspiration.

Bar codes (Figure 5B) also validate inspiration and expiration classes. A bar code represents the symbolization of a raw signal as a timeline where inspirations and expirations are colored accordingly to their classes. Bar codes reveal the dynamics of respiratory behaviors and their changes. For example, inspiration classes for control mice (WT) after physostigmine injection are almost unchanged. On the contrary, their expiration classes change significantly after a latent period. This dynamic is consistent with results in Nervo et al. (2019) where the mean frequency per minute of respiratory cycle decreases after the injection of physostigmine for control mice (WT). The frequency decrease corresponds to an increase in the duration of the post-inspiration pauses per min. Through the bar codes, it is possible to visualize the appearance of expiration classes 3 and 4 after injection with remarkable precision.

The inspiration and expiration classes have been constructed without prior knowledge of mice’s breathing behaviors. Nonetheless, the classes present differences that can be interpreted in terms of physiological modifications. For instance, some of the expiration classes represent post-inspiratory pauses. New inspiration classes have also been described, probably related to the motor controls dynamics during the active ventilation phase.

### 5.2 Classes reveal heterogeneity: an observation masked by the averaging of algebraic descriptors

Analyses on a small cohort can be biased if individual responses are heterogeneous. Unfortunately, it is often difficult to recognize this heterogeneity through some descriptors. The new symbolization, based on typical inspiration/expiration, the visualization and the quantification tools we proposed, offer perspectives on this critical issue in biology. For example, it is apparent on individual RC maps and bar codes that control mice (WT) present homogeneous respiration; the respiration cycle types are A0 and B0. After injection of AChE inhibitor, the RC maps and bar codes of control mice WT-1, 2, 6, 7 show that they follow the same evolutionary dynamics. Nevertheless, mice WT-3, 8 present different dynamics, and mice WT-4,5 died during the experiment. Thus, we can conclude that mice adapt differently to cholinesterase inhibition by physostigmine. In addition, the tests highlight changes that are significantly different.

**FIGURE 9:**
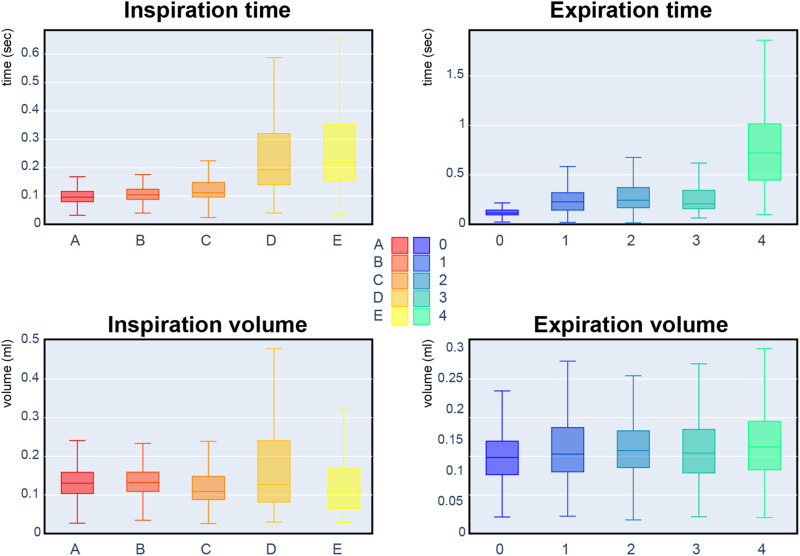
Box plots of the respiratory cycle descriptors: inspiration/expiration time and inspiration/expiration volume. Each box plot represents a referent sequence. A box represents the first quartile (Q1), median, and third quartile (Q3). The lower whisker corresponds to the minimum value observed, and the upper whisker is above the third quartile by 1.5 interquartile range (IQR: Q3-Q1).

We proposed in Boudinot et al. (2009) and Nervo et al. (2019) that mice with partial AChE deficiency were remarkably adapted to AChE deficit in the brain, autonomic nervous systems, and muscles. Indeed, the most frequent respiratory cycles before injection are composed with the inspiration of type A, B, C and the expiration of kind 0, 1, 2. Looking at Figure 9, these reference sequences share similar duration and volume. Therefore, it is impossible to differentiate the genotypes based on inspiration/expiration duration or volume.

The present study shows that the distributions of inspiration and expiration classes on AChE1iRR mice are similar. AChE1iRR mice do not have AChE in skeletal muscle. These mice show a high homogeneity of adaptation despite muscle weakness. In contrast, PRiMA mice, which have AChE deficiency in the brain and autonomic nervous systems, adapted well to AChE inhibition, but showed heterogeneous respiratory behavior. The heterogeneity is apparent in inspiration and expiration classes, which suggests the possibility of different respiratory behaviors to cope with AChE deficit in the nervous system. The cohort of ColQ mice also presents heterogeneity in respiratory behaviors, specifically for inspiration. As discussed, the inspiration of ColQ mice is characterized by types C and D. In contrast, the inspiration of other genotypes is characterized by types A and B. While ColQ and AChE1iRR mice have similar AChE deficiency in neuromuscular junctions, AChE1iRR mice adapt better than ColQ mice which also have AChE deficit in other tissues. This result suggests that AChE deficit in skeletal muscle is insufficient to affect these mice’s inspiration.

If the respiratory adaptations are different, it is not surprising that the consequences of the injection of physostigmine are so variable. Visualization of inspiration and expiration classes, either in RC maps or bar code, makes it possible to account for this diversity. After injection of physostigmine, the changes tend to affect inspiration in AChE1iRR and ColQ mice, whereas expiration is more affected in WT and PRiMA mice.

In summary, representing respiratory cycles by classes sharing similar shapes reveals a diversity of unsuspected respiratory behaviors that were not identifiable with descriptors deduced from the airflow. This rich information is synthesized in graphical representations highlighting how mice respond differently to cholinesterase deficits or inhibition.

### 5.3 Inspiration and expiration classes evoke distinct biological processes

Inspiration and expiration classes are defined without prior knowledge of underlying biological processes. Inspiration classes A and B represent a regular inspiration phase, while classes C and D represent an inspiration phase with a more or less significant pause. The pauses in category C are very short and always during inspiration; they probably correspond to a motor impairment during lung inflation (the main action of the diaphragm, a powerful muscle) or by a fine control of the glottis. The longer pauses of class D may occur during the air inflow and are probably similar, in nature, to class C. In contrast, the long pauses of Class E correspond to a sort of pause before the air enters the lungs. From a physiological point of view, these pauses could correspond to a delay in the glottis’s active opening, which is required to allow air to enter into the trachea. Two situations can lead to the glottis remaining closed: the cessation of muscle contractions that control the glottis opening or the spasm (cramp) of the muscles that control the closing of the glottis. Expiration class 0 represents a regular and probably passive phase of expiration. Classes 2, 3 and 4 start with a post-inspiratory pause whose duration increases progressively from category 2 to category 4. These post-inspiratory pauses are well described in the literature and appear in different physiological conditions. They appear when it is necessary to increase the air pressure in the lungs (short pauses) or as reflexes (long pauses), such as those resulting from inhaling molecules that irritate the upper airways (Dutschmann et al., 2014).

From these results we can conclude that inspiration and expiration classes learned from a subset of recordings selected from (Nervo et al., 2019) carry interpretable physiological meaning. It is important to note that these classes are specific to the experiment. For instance, applying our method to a set of signals presenting bronchoconstrictions will likely lead to classes differentiating the severity/variety of constrictions in a finer way than using the EF50 metric (Glaab and Braun, 2021).

### 5.4 Limits and future work

Our approach to analyzing respiratory signals is based on learning typical inspirations and expirations, called reference sequences. Currently, the number of referent sequences is arbitrarily set by the user. By doing so, the user chooses the degree of detail incorporated in the symbolization: adding referent sequences divides typical breathing behaviors into subgroups with minor variations. In that manner, reference sequences carry meaningful physiological information for the user. Nevertheless, choosing a good number of reference sequences can be complicated and time-consuming without knowledge of respiratory behavior. In such cases, several heuristics based on mathematical criteria exist to define the number of reference sequences automatically (Kodinariya and Makwana, 2013). In any case, these heuristics can be used as a starting point to properly define the number of clusters in light of the experiment objective.

In the current work, we limited ourselves to static descriptors of reference sequences (RC map, polar plot) and visual interpretation of the breathing behavior evolution over time (bar plot). Nevertheless, breathing behavior dynamics can also be quantified using the proposed symbolization of recordings and applying symbolic dynamics theory (Morse and Hedlund, 1938; Lind and Marcus, 2021). Symbolic dynamic theory has been developed to study how a system’s configurations change over time and how similar initial states can grow dissimilar.

During the experiment, we only symbolized recordings included in the training data set. By doing so, we guaranteed that the most common behaviors present in the recordings were taken into account during the learning step. We do not recommend symbolizing on other recordings as some typical behavior might be negelcted. In future work, we would like to investigate the use of a hierarchical clustering algorithm on a large data set composed of recordings with various experimental conditions. By doing so, we would like to create a universal referential of typical behaviors usable across experiments that can adapt to the level of detail required by selecting a symbolization directly from the hierarchy.

### 5.5 Prospective use

In this work, we have limited ourselves to plethysmograph signals recorded with DCP, but the method and it can easily be extended to head-out plethysmography (Bruggink et al., 2022) as well as to other biological systems. Indeed, our approach relies on accurate segmentation of plethysmograph signals, allowing relevant studies of inspiration and expiration. Any biological system which results in the recording of a cyclic signal can use our approach with proper segmentation. For instance, in the case of electrocardiogram signals, we could combine our approach with a heartbeat detection algorithm (Pan and Tompkins, 1985; Zong and Jiang, 2003) to detect and represent patterns of diseases like arrhythmias, heart attacks, cardiomyopathy, and coronary heart disease. Then, the symbolization of these signals could offer insightful information about the underlying dynamics of such diseases.

## Data Availability

The original contributions presented in the study are included in the article/Supplementary Material, further inquiries can be directed to the corresponding author.
